# P-1111. An investigation of the characteristics of and measures to prevent blood-borne occupational exposure among medical staff in a tertiary general hospital

**DOI:** 10.1093/ofid/ofaf695.1306

**Published:** 2026-01-11

**Authors:** Hao Hua, Jinlan Lin, Sheng Wu

**Affiliations:** Beijing Tsinghua Changgung Hospital, Beijing, Beijing, China (People's Republic); Beijing Tsinghua Changgung Hospital, Beijing, Beijing, China (People's Republic); Beijing Tsinghua Changgung Hospital, Beijing, Beijing, China (People's Republic)

## Abstract

**Background:**

Hospital workers face high risks of blood-borne occupational exposure , transmitting diseases like hepatitis and HIV. Standardized prevention effectively reduces exposure. Investigate exposure characteristics, causes, and protective measures among Chinese tertiary hospital staff through post-exposure surveys, aiming to optimize occupational safety strategies.

**Figure:**
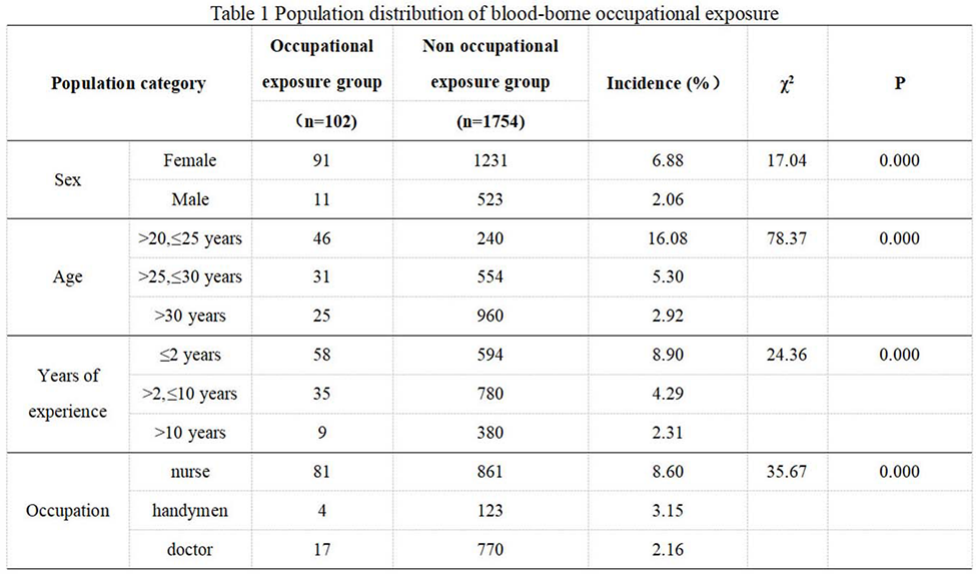

**Figure:**
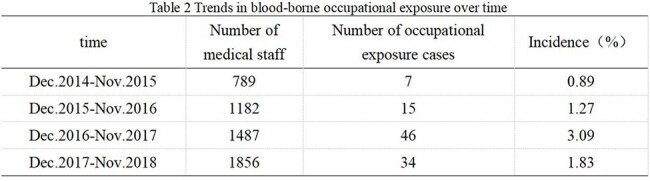

**Methods:**

The causes of blood-borne occupational exposure in a tertiary general hospital between December 1, 2014, and November 30, 2018 were retrospectively investigated. Data, including personnel information, incidence of occupational exposure, source of infection, and protective measures during exposure, were analyzed using SPSS 16.0.

**Figure:**
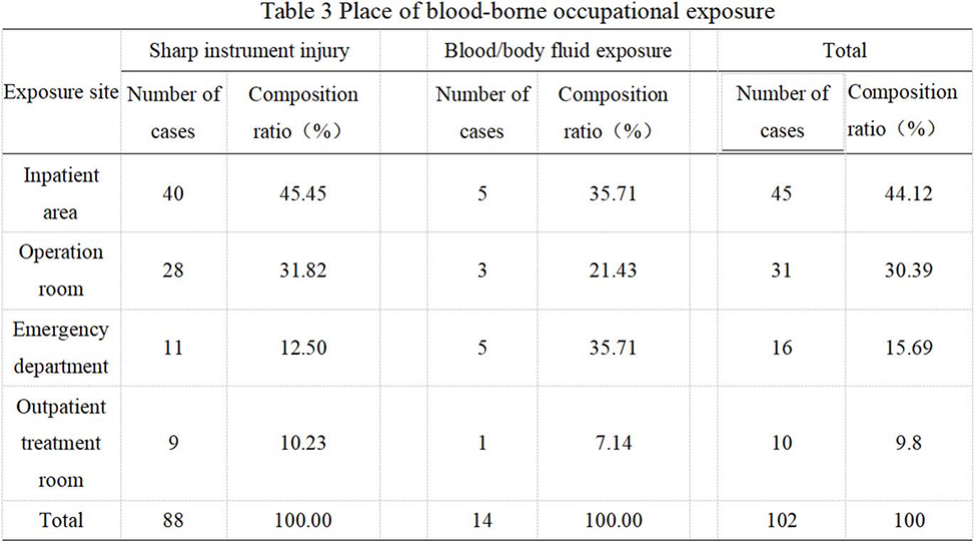

**Figure:**
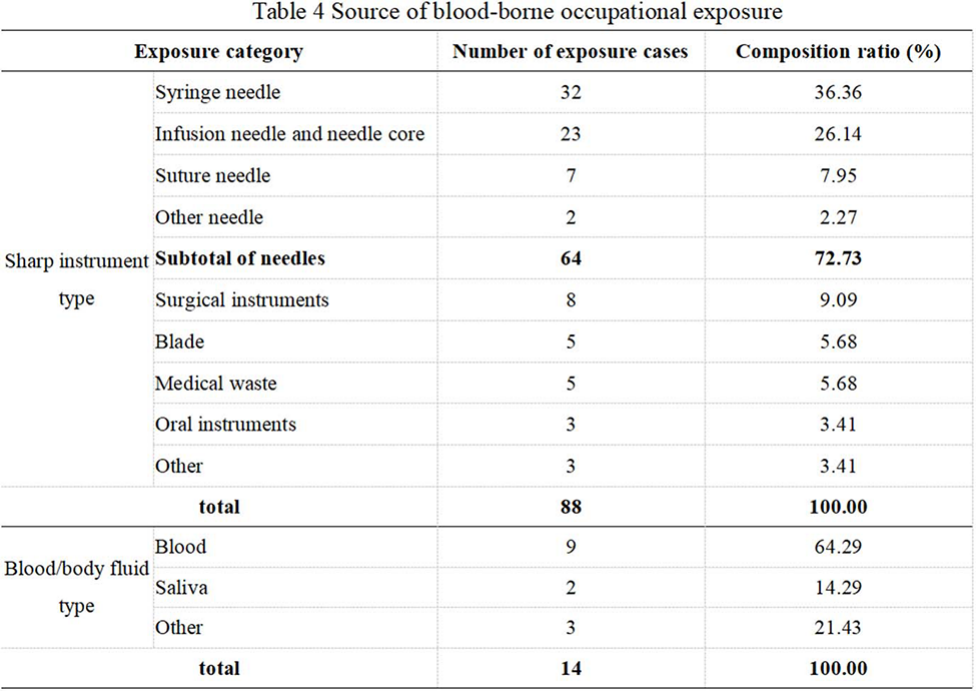

**Results:**

There were 102 cases of blood-borne occupational exposure in 4 years. Among these cases, 88 (86.3%) involved sharp instruments, and 14 (13.7%) involved blood and body fluids. The incidence in females (6.88%) was higher than that in males (2.06%). The proportion of cases involving younger personnel was greater than that involving older personnel (≤25 years old 16.08%, > 25 years old and ≤30 years old 5.3%, >30 years old 2.92%). The proportion of cases involving inexperienced personnel was greater than that involving experienced personnel (< 2 years 8.9%, > 2 and ≤ 10 years 4.29%, > 10 years 2.31%). There were significantly more nurses than support personnel and doctors (8.60%, 3.15%, 2.16%) (P< 0.001). The incidence of occupational exposure increased annually (0.89%, 1.27%, 3.09%, and 1.83%, respectively). Injuries involving sharp instruments are common in wards and operating rooms, with needlestick injuries being the most common (72.73%). The incidence of blood/body fluid exposure is 64.29% in wards and emergency rooms, most frequently due to blood spatter during puncture. Fifty-five patients (53.92%) were exposed to infectious factors. In 42 cases (47.73%), personnel were wearing gloves at the time of sharp instrument injury. In 7 cases (50%), personnel were wearing medical surgical masks at the time of blood/body fluid exposure.

**Conclusion:**

Personnel should be informed of the potential causes of blood-born occupational exposure (e.g., potential hazards) and receive instruction on the proper use of protective equipment.

**Disclosures:**

All Authors: No reported disclosures

